# Reproductive cycles in tropical intertidal gastropods are timed around tidal amplitude cycles

**DOI:** 10.1002/ece3.3166

**Published:** 2017-06-23

**Authors:** Rachel Collin, Kecia Kerr, Gina Contolini, Isis Ochoa

**Affiliations:** ^1^ Smithsonian Tropical Research Institute Balboa Republic of Panama; ^2^ Department of Biological Sciences University of Alberta Edmonton AB USA; ^3^ Long Marine Lab Department of Ecology and Evolutionary Biology University of California at Santa Cruz Santa Cruz CA USA

**Keywords:** egg mass, invertebrate, life histories, littorinid, moonsnail, phenology, predator avoidance, reproduction

## Abstract

Reproduction in iteroparous marine organisms is often timed with abiotic cycles and may follow lunar, tidal amplitude, or daily cycles. Among intertidal marine invertebrates, decapods are well known to time larval release to coincide with large amplitude nighttime tides, which minimizes the risk of predation. Such bimonthly cycles have been reported for few other intertidal invertebrates. We documented the reproduction of 6 gastropod species from Panama to determine whether they demonstrate reproductive cycles, whether these cycles follow a 2‐week cycle, and whether cycles are timed so that larval release occurs during large amplitude tides. Two of the species (*Crepidula* cf. *marginalis* and *Nerita scabricosta*) showed nonuniform reproduction, but without clear peaks in timing relative to tidal or lunar cycles. The other 4 species show clear peaks in reproduction occurring every 2 weeks. In 3 of these species (*Cerithideopsis carlifornica* var. *valida, Littoraria variegata*, and *Natica chemnitzi*), hatching occurred within 4 days of the maximum amplitude tides. *Siphonaria palmata* exhibit strong cycles, but reproduction occurred during the neap tides. Strong differences in the intensity of reproduction of *Cerithideopsis carlifornica*, and in particular, *Littoraria variegata*, between the larger and smaller spring tides of a lunar month indicate that these species time reproduction with the tidal amplitude cycle rather than the lunar cycle. For those species that reproduce during both the wet and dry seasons, we found that reproductive timing did not differ between seasons despite strong differences in temperature and precipitation. Overall, we found that most (4/6) species have strong reproductive cycles synchronized with the tidal amplitude cycle and that seasonal differences in abiotic factors do not alter these cycles.

## 1 INTRODUCTION

Evolutionary ecological theory predicts that reproductive investment responds to diverse ecological drivers so that it is timed and partitioned to optimize offspring survival. In many marine and terrestrial organisms, reproduction is timed to coincide with seasonal increases in resource availability and thus to maximize offspring growth and survival. For example, many birds time reproduction to coincide with maximum seasonal abundances of insects (Thomas, Blondel, Perret, Lambrechts, & Speakman, 2001), or zooplankton and fishes (Durant, Anker‐Nilssen, & Stenseth, 2003; Regehr & Montevecchi, 1997; Suryan, Irons, Brown, Jodice, & Roby, 2006). Reproduction may also be timed to coincide with the availability of ephemeral habitats necessary for embryonic or larval development. Insects and amphibians that rely on seasonally available pools of water for larval development time reproduction with the wettest times of the year (Shine & Brown, 2008), and parasitoids time reproduction to coincide with the availability of hosts (Hood et al., 2015). Reproduction may also be timed to minimize mortality due to harsh environmental conditions, or predation on mothers or offspring (Christy, 2011). For example, many marine organisms release larvae or spawn during the night to avoid visual predators (Christy, 2011). It has been suggested that predation may drive the evolution of synchrony of emergence of cicadas, annual mass spawnings of corals, and synchronized reproduction of sea turtles as predator‐swamping strategies (Ims, 1990; Williams, Smith, & Stephen, 1993).

Among marine organisms, intertidal crabs provide the best‐understood example of the adaptive optimization of reproductive timing (Christy, 2003, 2011; Christy & Stancyk, 1982; Morgan & Christy, 1994, 1995, 1997; Skov et al., 2005). More than 80 species of crabs have been shown to time larval release to coincide with times when predation by visual predators is lowest (i.e., nighttime) and when tidal currents are most likely to quickly move larvae away from shallow water (Christy, 2011). In general, tidal currents are greatest when the tides have the largest amplitude. As tidal amplitude follows a lunar or semilunar cycle (depending on the location), this produces a lunar or semilunar cycle in the timing of larval release (Christy, 2003, 2011; Christy & Stancyk, 1982). Describing biological cycles as “lunar” or “semilunar” simply reflects the duration of the cycle and should not be read to imply a causal relationship with the lunar phases, as reproductive cycles for a number of crabs show precise matching to details of the tidal amplitude cycle, adjusting reproduction so that larvae are released during the tides with the largest amplitude in the month, regardless of whether these occur during the full or new moon (Skov et al., 2005). In addition, some species can adjust the timing of mating to compensate for the temperature dependence of development and ensure that hatching occurs during the optimal time regardless of temperature (Kerr, 2015; Kerr, Christy, Collin, & Guichard, 2012; Kerr et al., 2014). Overall, intertidal crabs follow the same cycles regardless of habitat or taxonomic group, while the exceptions are explained by particular features of the larvae thought to reduce predation in other ways, or constraints on the ability to release larvae at the optimal time imposed by the habitat (Morgan, 1996a,b; Morgan & Christy, 1994, 1995).

Semilunar or lunar cycles of various kinds have been reported only occasionally for other groups of iteroparous marine invertebrates. Those species for which cycles have been commonly reported are primarily subtidal and include lunar cycles in sea urchins, where synchronized reproductive timing is thought to ensure fertilization success (e.g., Lessios, 1991; Mercier, Ycaza, & Hamel, 2007; Pearse, 1975) and lunar release of larvae from brooding corals (e.g., Richmond & Jokiel, 1984; Tanner, 1996). It is surprising that semilunar or lunar reproductive cycles have been reported for so few intertidal invertebrates, because avoidance of visual predators by offshore advection of larvae could benefit species other than crabs. In addition, the lives of intertidal animals revolve around the progression of the tides. The tidal amplitude and the time of the tide relative to the diel cycle affect all environmental variables in this habitat, thus influencing activities including foraging, access to mates, and molting of a wide range of organisms (Weisberg, Whalen, & Lotrich, 1981; Iwasaki, 1995; Tran et al., 2011; reviewed in Naylor, 1999). Therefore, it seems intuitively likely that the reproduction of intertidal invertebrates could be shaped by factors other than offshore advection of larvae that could also result in reproductive cycles synchronized with the tides.

We surveyed the reproductive timing of 6 species of tropical intertidal gastropods with diverse natural histories to determine whether reproduction is synchronized among individuals in the population and whether this synchrony follows a regular cycle relative to the lunar or tidal amplitude (semilunar) cycle. We aimed to answer the following questions for each species: (1) Does reproduction follow a synchronized monthly or bimonthly cycle? (2) If reproduction is periodic, is the cycle more closely related to the lunar or tidal amplitude cycle? (3) If present, are reproductive cycles timed to release larvae into the water column during spring tides, as they are in crabs? and (4) Is the timing of reproductive cycles altered by seasonal changes in environmental conditions. Cycles with timing similar to those reported for crabs would be consistent with the idea that the same risk factors drive reproductive timing in gastropods. Cycles with different timing would point to different selective factors shaping reproductive synchrony. A lack of synchrony would suggest that the timing of egg deposition and/or hatching relative to lunar or tidal amplitude cycles are not selectively important.

## 2 METHODS

**Figure 1:**
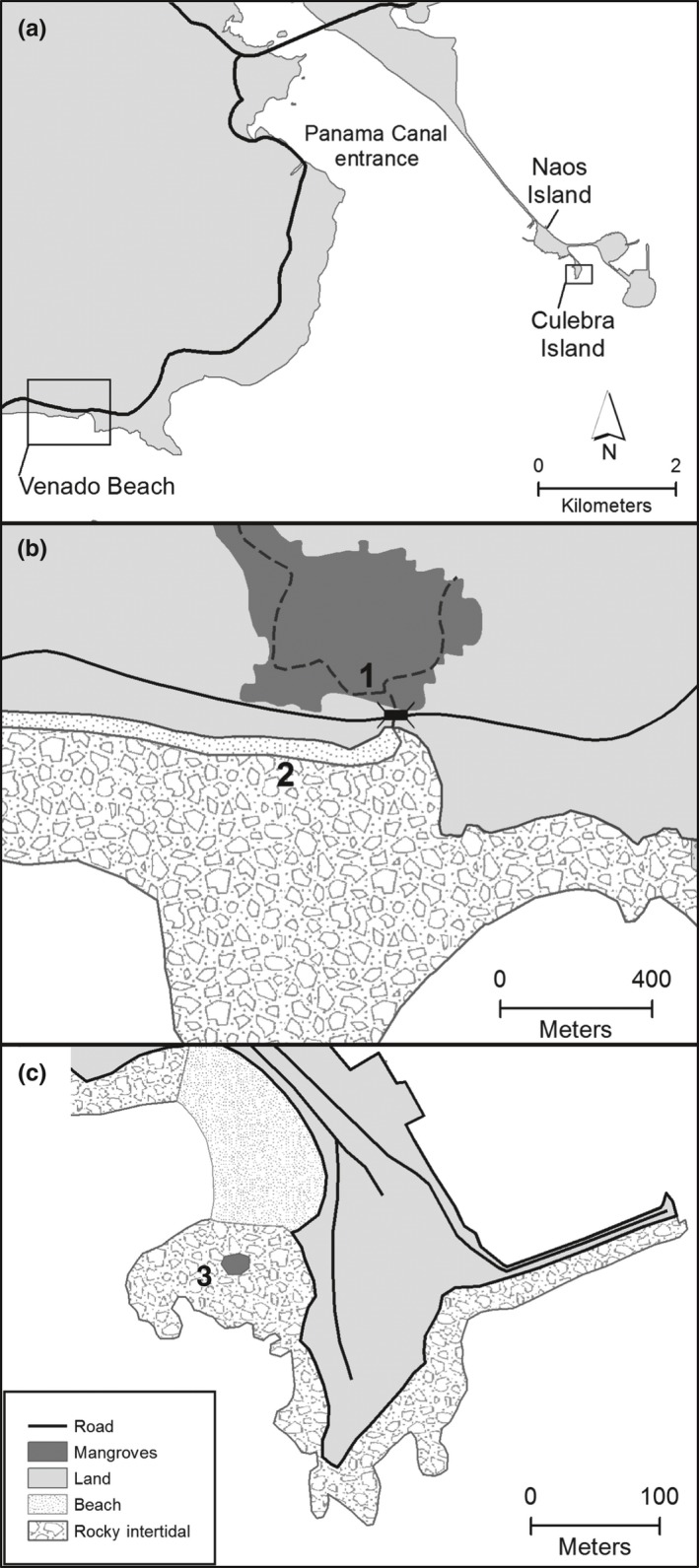
Maps of the collecting locations along the Pacific coast of Panama. (a) An overview of the area around the entrance to the Panama Canal on the outskirts of Panama City. (b) Venado Beach 8.892°N, 79.597°W, showing the distribution of habitats at low tide. (1) indicates the mangroves where *Cerithideopsis californica* and *Littoraria variegata* were collected. (2) indicates the sandy beach where masses of *Natica chemnitzi* and broods of *Crepidula* cf. *marginalis* were observed. (c) Punta Culebra, 8.911°N, 79.529°W on Culebra Island, showing the distribution of habitats at low tide. (3) the location where masses of *Siphonaria palmata* and *Nerita scabricosta* were observed

The 6 snail species were studied at sites where they are abundant in the intertidal of the Bay of Panama. Two species, the nerite *Nerita scabricosta* and pulmonate limpet *Siphonaria palmata*, were studied in the rocky intertidal of Culebra Island, near Panama City at the entrance to the Panama Canal (Figure 1a,c). Four other species (*Cerithideopsis californica*,* Natica chemnitzi*,* Crepidula* cf. *marginalis*, and *Littoraria variegata*) were studied at Playa Venado, near the town of Veracruz, 8 km from Culebra Island (Figure 1a,b; Collin & Ochoa, 2016). Tides in the Bay of Panama are semidiurnal with an average amplitude of 3.9 m and a maximum amplitude of 6.9 m. The largest amplitude, or spring, tides of the 14.8‐day amplitude cycle occur consistently 2–3 days after the full and new moons, but the largest spring tides of the 29.5‐day lunar cycle may occur after either a full or a new moon. We used the nighttime ebb amplitude, which was calculated as the difference between the height of the nighttime high tide and the height of the following low tide. In general, high tides on large amplitude (spring) tides occur between 5:00 and 7:00 in the morning and evening, while the high tides on the small amplitude (neap) tides occur around noon and midnight. The tight relationship between the tidal amplitude and the time of day of the tides means that a number of abiotic factors vary in concert with the amplitude cycle, making it difficult to attribute causality of semilunar cycles to any specific abiotic factor without employing experimental manipulations.

The Bay of Panama experiences distinct seasonal variation in precipitation, wind patterns, and upwelling of cold waters causing seasonal changes in temperature and salinity. The dry season, when upwelling occurs, generally extends from mid‐December to early May. Late May to early December is characterized by frequent rain and relatively warm and constant sea surface temperature (Collin & Ochoa, 2016; Robertson & Collin, 2015). These seasonal changes in abiotic factors affect the timing and intensity of reproduction of several organisms. Hence, we compared the timing of reproduction during the wet and dry season to explore the potential for seasonal changes in abiotic factors to alter timing.

### 2.1 Study species and methods

To determine whether reproductive events of these snail species follow a cycle, we surveyed the abundance of egg masses, or the timing of egg deposition or release of larvae, depending on the species. Each species was studied for several months, and for four of these species, this was done in parallel with a study of seasonal differences in egg mass abundance and offspring size (Collin & Ochoa, 2016). We determined the timing of reproductive events relative to the tidal amplitude and lunar cycles in different ways based on the idiosyncrasies of the natural history of each species.

#### 2.1.1 *Littoraria variegata*

*Littoraria variegata* live on the trunks and lower branches of mangrove trees in the mixed mangrove forest (*Avicenia germinans*,* Avicenia bicolor*,* Laguncularia racemosa* and *Rhizophora* sp.). They release pelagic egg capsules each of which surrounds a single egg. Nine to 36 (mean 19.5) female *Littoraria* were collected 1–2 times a week. They were maintained in the laboratory, in the dark, in a temperature‐controlled incubator at 28°C in 50‐ml Falcon tubes half‐filled with seawater. Every day the water was emptied from the tubes, and the presence or absence of pelagic egg capsules was recorded. The % of females releasing capsules each day was used in the analyses. The data from this species are similar to those generated by studies designed to determine whether cycles can be maintained in the absence of environmental cues (e.g., Forward, 1987). Data were collected between October 2013 and August 2014.

#### 2.1.2 *Crepidula* cf. *marginalis*

Broods of the slipper limpet *Crepidula* cf. *marginalis* were studied on the beach at Playa Venado, which is a mix of muddy sand and rock rubble. *Crepidula* cf. *marginalis* are abundant on small rocks, at the same tidal height as the moonsnail egg masses (see below). Slipper limpets brood egg masses between their neck, propodium, and the substrate. Every Monday, Wednesday, and Friday 10–20 large (>9 mm) *Crepidula* cf. *marginalis* that were paired with a male were removed from the rocks, and the presence or absence of a brood was recorded. Mature broods turn brown when they are within 1–2 days of hatching, and the presence of brown broods was scored as a proxy for hatching and used in the statistical analyses. These snails were surveyed between November 2013 and August 2014.

#### 2.1.3 *Cerithideopsis californica*

*Cerithideopsis californica* var. *valida* (referred to subsequently as *C. californica*) were studied in the high intertidal in the mixed mangrove forest (*Avicenia germinans*,* Avicenia bicolor*,* Laguncularia racemosa*, and *Rhizophora* sp.). *Cerithideopsis californica* inhabit the mudflat and the base of the mangrove trees and deposit gelatinous egg strings on the surface of the mud (Miura, Frankel, & Torchin, 2011). Three 1 m × 1 m permanent quadrats were installed on the mud at the base of mangrove trees. Every Monday, Wednesday, and Friday, we marked each new egg mass with labeled sticks and removed the sticks when the masses had hatched. The number of sticks deployed and retrieved each day was recorded. Deployment and removal of sticks were used as proxies for egg deposition and hatching, respectively, and used in the statistical analyses. These actual events occurred <2 days prior to the reported event. Data were collected between November 2013 and August 2014.

#### 2.1.4 *Nerita scabricosta*

*Nerita scabricosta* deposit small blister‐shaped capsules in small tide pools in the high rocky intertidal of Isla Culebra (Collin, Roof, & Spangler, 2016), slightly above those in which *S. palmata* deposit egg masses. Photographs of two quadrats in 15 pools were taken every Monday, Wednesday, and Friday. The number of bright white, newly deposited egg capsules was counted from each photograph to record deposition. Newly hatched capsules were counted as those where a circular scar occupied a location that had a capsule the previous day. The actual events occurred <2 days prior to the reported proxy for the event. Data were collected between August and October 2014.

#### 2.1.5 *Natica chemnitzi*

Egg masses of the moonsnail *Natica chemnitzi* were studied on the beach at Playa Venado, which is a mix of muddy sand and rock rubble. *Natica chemnitzi* deposit sand‐covered egg collars typical of moonsnails on top of the sand or muddy sand in the high midintertidal (Collin & Ochoa, 2016). Every Monday, Wednesday, and Friday, we placed three 100 m × 2 m band transects perpendicular to the coast in the zone where *Natica* egg masses occur. As masses could not be followed individually, but clear cycles were present in this species, we predicted peak hatching times for each tidal amplitude cycle and analyzed them as reproductive events. The peak in hatching should occur approximately *n*/2 days after the peak number of masses and *n* days after the peak in egg deposition, where *n* is the time to hatching.

All the masses in each transect were counted between January 2013 and August 2014.

#### 2.1.6 *Siphonaria palmata*

*Siphonaria palmata* deposit gelatinous masses in small tidal pools in rocks in the high midintertidal of Isla Culebra. Every Monday, Wednesday, and Friday, we counted the number of egg masses in twenty 7 cm × 7 cm quadrats. As the loose ribbons could not be tracked individually, but clear cycles were present in this species, we predicted peak hatching times for each tidal amplitude cycle and analyzed them as reproductive events. The peak in hatching should occur approximately *n*/2 days after the peak number of masses and *n* days after the peak in egg deposition, where *n* is the time to hatching. Data were collected between September 2015 and June 2016.

### 2.2 Statistics

Most previous studies of invertebrate reproductive cycles plot events relative to time and draw conclusions by visual inspection of the tidal and lunar phases relative to the reported events (see section 4). These graphs are intuitively easy to interpret, but they make detection of small differences difficult, and conclusions drawn this way are limited to indicating which general part of the cycle seems to fit with peaks in the data. We used circular statistics to quantify how reproduction was clustered relative to the semilunar tidal amplitude cycle and the lunar cycle. Reproductive events were plotted on circular plots as an angle in degrees relative to the tidal amplitude or lunar cycle in R using the package GGplot 2 (Wickham, 2009). Each day in the cycle of interest was converted to degrees by dividing 360° by the number of days in the cycle. If reproductive peaks occur at the same point in the cycle across multiple cycles, the points will appear in a cluster covering only a few degrees of the circle. We visually inspected the data plotted on circular graphs representing the ~15‐day tidal amplitude cycle, the ~29‐day lunar cycle, and the ~monthly cycle of larger and smaller spring tides (two tidal amplitude cycles) with the day of the maximum amplitude nocturnal tide, the day of the full moon, and the day of the maximum amplitude tide of a month (largest spring tide) at 0°, respectively.

Because a 2‐week cycle was evident for most species, we used this tidal amplitude cycle to calculate descriptive circular statistics and tested for clustering of events using the CircStats2010d package in MATLAB (Berens, 2009). Mean angle, resultant vector length (R, a measure of dispersion or synchrony that ranges from 0, randomly scattered, to 1, identical values), and circular standard deviation were used to quantify the timing and synchrony of events relative to the tidal amplitude cycle. We used the Omnibus test for circular uniformity to determine whether events were unevenly distributed around the circle for each of the 6 species (Berens, 2009; Zar, 2010). A *p*‐value of <.05 indicates a significant difference from a uniform distribution of events. For those species with moderate to high synchrony (*R* > 0.4), we used a v‐test to determine whether reproductive events occurred near the timing of the maximum tides. The v‐test for circular uniformity tests between the null hypothesis of a uniform distribution and an alternate hypothesis of a nonuniform distribution with a known mean direction. To test whether events occurred near the large amplitude tides, we tested an alternate hypothesis with an angle of 0°.

#### 2.2.1 Lunar versus tidal amplitude cycles

The largest spring tides of a lunar month are sometimes associated with the new moon and sometimes with the full moon. To determine whether snails with semilunar cycles time reproduction with the phases of the moon or with the size of the spring tides, we plotted the timing of reproduction during the month relative to the largest spring tides (0°) and relative to the lunar phase (full moon = 0°). Plotting the data this way will produce 1 cluster if they follow a 1‐month cycle and 2 clusters of points ~180° apart if the events follow the 2‐week cycle. If more reproductive events occur during one phase of the moon compared to the other, we would expect more points in one cluster on the circular graph or for the points in one cluster to extend further from the center than the points in the other cluster. The same logic applies when the data are mapped relative to the 1‐month amplitude cycle, which includes both the large and small spring tides of a month. If the snails are targeting the largest amplitude tides of the month, more points will occur around 0° on the tidal amplitude graph, rather than two equal clusters at 0° and 180°. If the snails are targeting a specific phase of the moon, points will also occur in one large cluster and one smaller cluster. For the species with bimodal patterns in reproduction relative to the monthly or lunar cycle, we tested for a difference in intensity of reproduction between the two hemispheres of the circle using binomial tests. We defined the two hemispheres by dividing them with a line perpendicular to the axis running through the clusters. A significant difference indicates that the species preferentially reproduces during one half of the lunar or monthly tidal amplitude cycle.

#### 2.2.2 Impact of environmental conditions on timing

To determine whether seasonal variation in abiotic conditions alters the timing of reproduction relative to the tidal amplitude cycle, we compared the average timing of events relative to the tidal amplitude cycle in the dry season (December–May) to that of the wet season (late May–early December).

## 3 RESULTS

### 3.1 Overview of results

**Figure 2:**
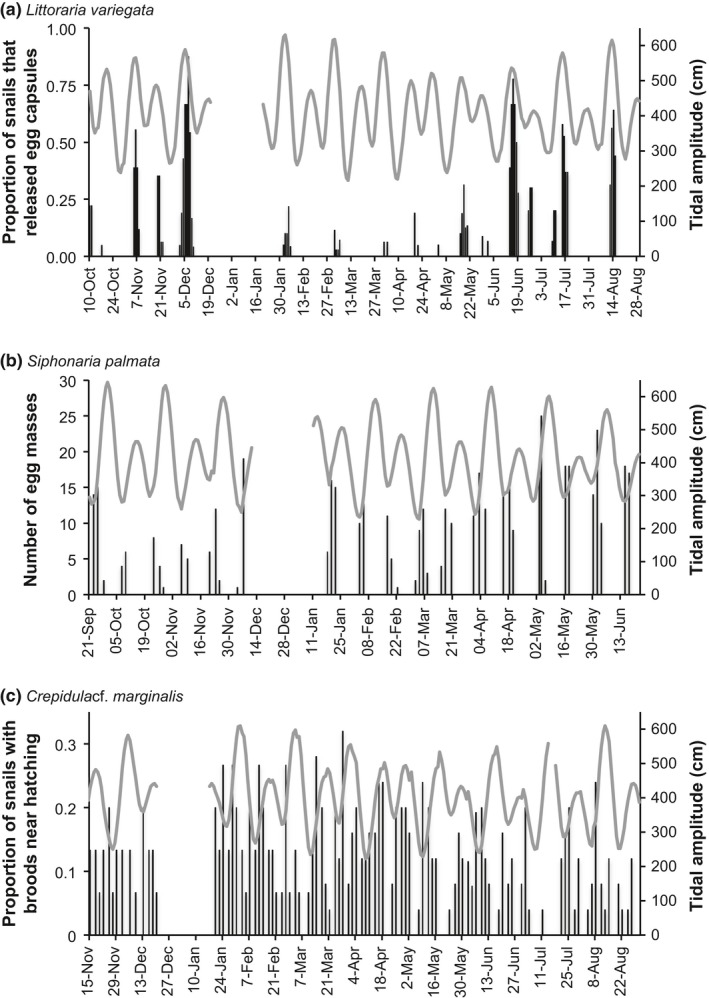
Number of masses through time for (a) *Littoraria variegata*, which shows reproductive peaks coincident with large amplitude tides (October 2013–August 2014) proportions based on an average of 19.5 animals per collection, (b) *Siphonaria palmata*, which shows reproductive peaks following the neap tides (September 2015–June 2016), and (c) *Crepidula* cf. *marginalis*, which does not show clear reproductive cycles (November 2013–August 2014). Note that gaps in the tidal amplitude time series indicate periods when sampling did not occur (ex. *C*. cf. *marginalis*: December 21–January 16 and July 15–17) and that the time periods are not the same for the three subplots, so they cannot be compared directly vertically

**Figure 3:**
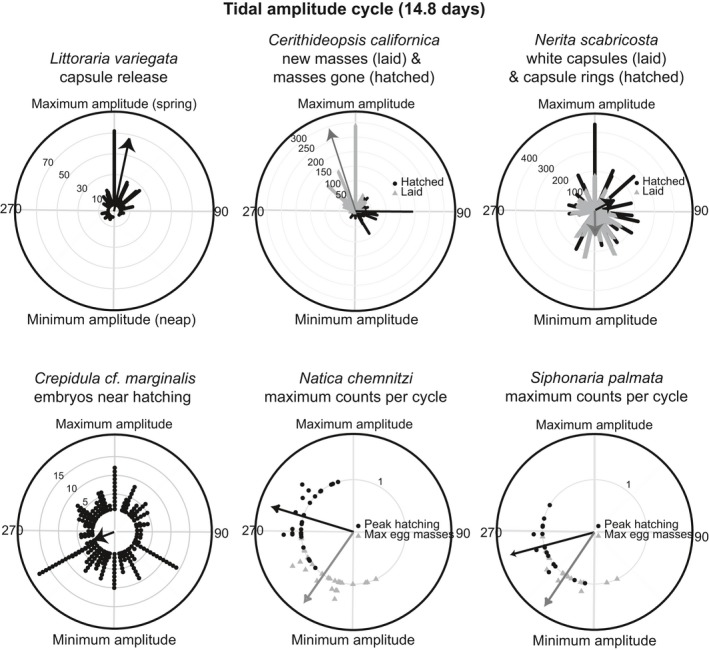
Circular plots of timing of reproductive events relative to the ~15‐day tidal amplitude cycle. Angles around the circumference of the circle represent the tidal amplitude cycle in days, with 0° representing the day of the maximum amplitude tide. Black dots and gray triangles represent individual occurrences of reproduction (hatching, release of larvae, presence of egg masses). For *Natica chemnitzi* and *Siphonaria palmata,* the gray triangles represent the dates of peak mass occurrence for each cycle and the black dots represent the predicted peak hatching for each cycle. The arrow represents the average timing of the reproductive events. The length of the arrow is scaled to the descriptive statistic R (resultant vector length), a measure of synchrony of events (the more synchronous the events, the longer the arrow). Summary circular statistics are provided in Tables 2, 3

**Figure 4:**
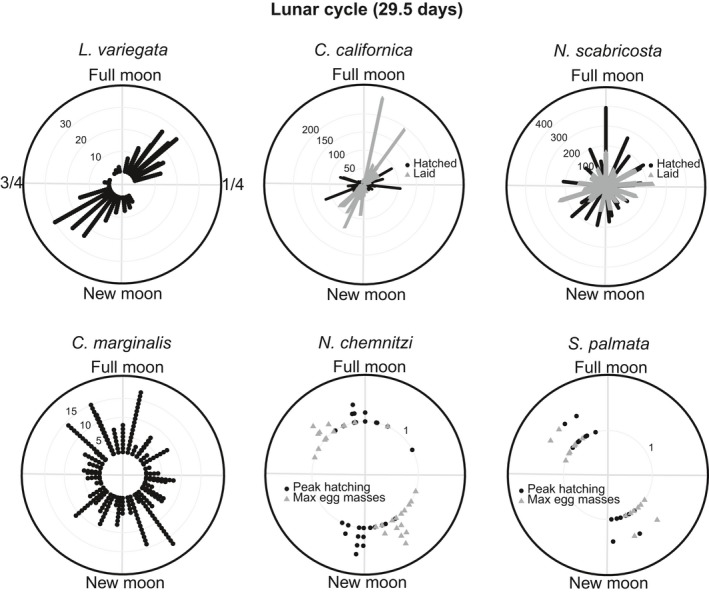
Circular plots of timing of reproductive events relative to the lunar cycle (~29 days). Angles around the circumference of the circle represent the lunar cycle in days, with 0° representing the day of the full moon and ~180° representing the day of the new moon. Black dots and gray triangles represent individual occurrences of reproduction (hatching, release of larvae, presence of egg masses). For *Natica chemnitzi* and *Siphonaria palmata,* the gray triangles represent the dates of peak mass occurrence for each cycle and the black dots represent the predicted peak hatching for each cycle. Summary statistics such as average timing were not calculated as the results are bimodal for most species when plotted relative to the full lunar cycle

**Figure 5:**
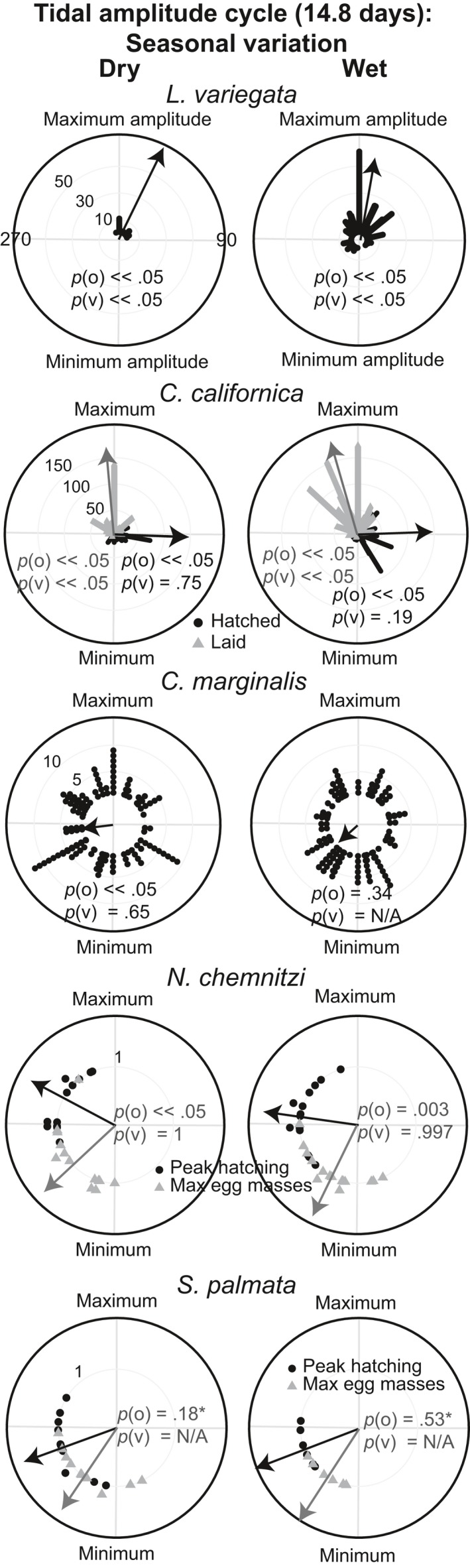
Circular plots of timing of reproductive events relative to the tidal amplitude cycle separated by season. The dry season occurs from mid‐December to early May, the wet season occurs from mid‐May to early December. Angles around the circumference of the circle represent the tidal amplitude cycle in days, with 0° representing the day of the maximum amplitude tide. Black dots and gray triangles represent individual occurrences of reproduction (hatching, release of larvae, presence of egg masses). For *Natica chemnitzi* and *Siphonaria palmata,* the gray triangles represent the dates of peak mass occurrence for each cycle and the black dots represent the predicted peak hatching for each cycle. The arrow represents the average timing of reproductive events. The length of the arrow is scaled to the descriptive statistic R (resultant vector length), a measure of synchrony of events (the more synchronous the events, the longer the arrow). *p*‐values for the omnibus test for circular uniformity (*p*(o)) and the v‐test (*p*(v)), which tests whether clustering of events are timed with a specified angle (0°) are shown on the figure (*p*(v) < .05 indicates significant clustering around the maximum amplitude tide. If the *p*‐value for the omnibus test is >.05, the timing relative to a specific angle cannot be tested (*p*(v) = N/A). *Nonsignificant *p*‐values for the omnibus tests for *S. palmata* are due to small sample sizes

**Figure 6:**
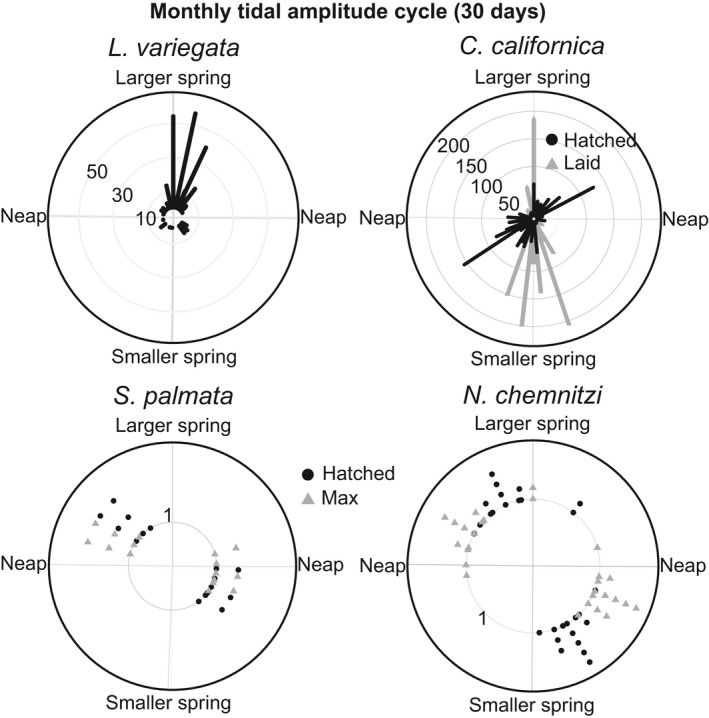
Circular plots of reproductive events for the 4 species which show clear synchrony relative to ~monthly cycle of larger and smaller spring tides (two tidal amplitude cycles). The circle represents the time in days between the largest spring tide of a month (0°) and the largest spring tide of the following month, 180° represents the day of the smaller spring tide, and 90° and 270° are the approximate timing of the neap tides. Reproductive events of *L. variegata* plotted relative to two tidal amplitude cycles (~1 month) show a clear difference in the number of females releasing larvae on the larger versus smaller magnitude spring tides. *C. californica* shows a less obvious, but significant, propensity for reproducing during the smaller spring tides. The other 2 species do not show a difference between the two tidal amplitude cycles

The duration of development varies from 4 to 28 days among the species studied (Table 1). For all six species, the omnibus test rejected a uniform distribution of reproductive events across the tidal amplitude cycle (omnibus test: *p* < .05, Figures 2, 3, Table 2). However, only 4 species (*L. variegata*,* C. californica*,* N. chemnitzi*, and *S. palmata*) showed clear cycles with periods of reproductive activity separated by periods in which reproduction did not occur (Figures 2, 3). When data from these 4 species were plotted relative to the lunar cycle, they exhibited a bimodal pattern of reproduction, indicating that they reproduce twice per lunar month, rather than targeting one particular phase of the lunar cycle (Figure 4). Three species with distinct cycles (*C. californica*,* N. chemnitzi*, and *S. palmata*) reproduce regularly during both the wet and dry seasons. None showed a change in timing or synchrony between the wet and dry seasons (Figure 5). The 4 species with strong reproductive synchrony showed different patterns in the timing of reproductive activity relative to the tidal amplitude cycle (Table 3, Figure 3). In *L. variegata*,* N. chemnitzi,* and *Cerithideopsis californica*, egg deposition or larval release occurred during the half of the tidal amplitude cycle with the largest amplitudes (i.e., between 270° and 90°). Egg deposition for *N. chemnitzi*, and both egg deposition and hatching of *S. palmata* occurred during the half of the cycle with the smallest amplitude tides. Of these four species, *L. variegata* and *C. californica* showed differences in reproduction between the two events that occurred each month. For *L. variegata*, virtually all events occurred during the larger of the two spring tides (Figure 6).

**Table 1:** Reproductive characteristics of the six species of Panamanian gastropods in this study

Species	Site	Habitat	Tidal height^a^ (m)	Egg mass	Reproductive season^b^	Days to hatch (*n*)^c^
*Littoraria variegata*	Playa Venado	Mangrove trees	7–10	Pelagic capsules^d^	Wet season	2–3 days (10)
*Natica chemnitzi*	Playa Venado	Sandy beach	3	Benthic egg collars	All year	5–6 days
*Cerithideopsis californica*	Playa Venado	Base of mangroves	4	Gel mass	Wet season	4 days (89)
*Siphonaria palmata*	Isla Culebra	Rock pools	4.25	Gel mass	All year	3 days (10)
*Nerita scabricosta*	Isla Culebra	Rock pools	4.5	Blister capsules	Wet season	28 days
*Crepidula* cf. *marginalis*	Playa Venado	Rock rubble	3	Brooded capsules	All year	9–10 days

Data from: Collin (2012), Collin and Ochoa (2016), Collin et al., 2016, and this study.

a Height above mean low tide.

b Wet season is May–December.

c The number of days for egg production to hatching.

d Tiny lens‐shaped capsules are released into the water column.

**Table 2:** Summary circular statistics and tests for synchrony of reproductive events

Species	Measure	*N*	Mean angle (°)	Resultant vector length (R)	Omnibus test for uniformity (*p*)
Peak release/hatching—high synchrony					
*Littoraria variegata*	Capsule release—Laboratory	331	12	0.70	<.001
*Cerithideopsis californica*	New masses (laid)—Field	1,268	347	0.79	<.001
*Cerithideopsis californica*	Masses gone (hatching)—Field	1,050	90	0.60	<.001
*Natica chemnitzi*	Projected peak hatching per cycle^a^—Field	28	287	0.82	<.001
*Siphonaria palmata*	Projected peak hatching per cycle^a^—Field	16	255	0.85	<.001
Peak release/hatching—low synchrony					
*Nerita scabricosta*	Capsule hatched (ring)—Field	4,174	62	0.15	<.001
*Nerita scabricosta*	Capsule deposition (white)—Field	2,788	179	0.19	<.001
*Crepidula* cf. *marginalis*	Embryos near hatching (brown)—Field	270	249	0.15	<.001

The mean angle is the average timing of events in degrees relative to the tidal amplitude cycle (1 day = ~24° and 0° is the point of maximum tidal amplitude). *R*, the resultant vector length, is a measure of synchrony of timing of events and ranges from 0 (no clustering) to 1 (all events happening at the same relative angle). The Omnibus test for circular uniformity tests whether events are uniformly distributed around the circle. A *p*‐value of <.05 indicates nonuniformity but does not necessarily indicate synchrony or a 14‐day cycle.

a For *N. chemnitzi* and *S. palmata*, hatching timing was estimated by adding one half of the development time to the timing of the maximum count of egg masses for each tidal amplitude cycle. Circular statistics were then calculated based on projected peaks of hatching per cycle.

**Table 3:** Circular statistics and tests for associations between peak release and spring tides

Species	Measure	*N*	Mean angle (CI) (°)	v‐test relative to 0° (*p*)
Significantly clustered around 0°—high and moderate synchrony				
*Littoraria variegata*	Capsule release	331	12 (6–17)	**<.001**
*Natica chemnitzi*	Projected peak hatching^a^	28	287 (272–302)	**.035**
*Cerithideopsis californica*	New masses (laid)	1,268	347 (344–349)	**<.001**
Not clustered around 0°—high synchrony				
*Siphonaria palmata*	Projected peak hatching^a^	16	255 (235–275)	.897
*Cerithideopsis californica*	Masses gone (hatching)	1,050	90 (85–93)	.396

The mean angle is the average timing of events in degrees relative to the tidal amplitude (1 day = ~24° and 0° is the day of the maximum tidal amplitude) cycle. The 95% confidence interval (CI) is provided as the lower and upper confidence interval limits. The v‐test tests for circular uniformity relative to a predefined angle, in this case 0° (representing the maximum amplitude tide). Rejection of the null hypothesis of circular uniformity indicates that the events are significantly clustered near 0°. A significant omnibus test (obtained for all species; Table 1) and a nonsignificant v‐test indicate that events are significantly clustered, but that the average timing of events differs significantly from 0°. Significant *p*‐values are represented in bold, and species are categorized as in Table 2.

a For *N. chemnitzi* and *S. palmata*, hatching timing was estimated by adding one half of the development time to the timing of the maximum count of egg masses for each tidal amplitude cycle. Circular statistics were then calculated based on projected peaks of hatching per cycle.

#### 3.1.1 *Littoraria variegata*

*Littoraria variegata* larvae hatch 2.2 days (*SD* = 0.42; *n* = 10) after capsule release at 28°C in the laboratory. Egg capsule releases were significantly clustered (Omnibus test *p* < . 05) with high synchrony (*R* = 0.70, Figures 2, 3). The average timing of capsule release was less than 1 day after the maximum amplitude tide (12°) and was not significantly different from the timing of the maximum amplitude tide (v‐test <0.05, Tables 2, 3). Intensity of reproduction decreased substantially during the dry season, but the average timing of release did not differ significantly from that of the wet season, ~1 day after the maximum amplitude tide (Figure 5). Reproduction occurred twice per lunar month, peaking approximately 3 days after the full and new moons (Figure 4). Although this species reproduces after both the new and full moon phases, with slightly higher reproduction during the full moon (binomial test: *p* = .048), it demonstrates strong modulation of intensity of reproduction with the size of the spring tides. Nearly all females released egg capsules during the larger spring tides of a lunar month regardless of lunar phase (Figures 2, 4, 6, binomial test: *p* ≪ .001). This indicates that tidal amplitude is more important for this species than is the lunar phase.

#### 3.1.2 *Crepidula* cf. *marginalis*

Previous studies have shown that development of *Crepidula* cf. *marginalis* embryos takes 9–10 days (Collin, 2012). Brown eggs (near hatching) of *Crepidula* cf. *marginalis* were observed throughout the tidal amplitude cycle, and synchrony was very low (*R* = 0.15). Nevertheless, the Omnibus test indicated that releases were nonuniform (*p* < .05), with average timing ~4–5 days before the maximum amplitude tide (Figures 2, 3, Tables 2, 3). No seasonal difference in synchrony or lunar patterns of reproduction was observed (Figures 4, 5).

#### 3.1.3 *Cerithideopsis californica*

Field observations of marked egg masses of *C. californica* showed development times of 4.0 days (*SD* = 1.7; *N* = 90). Egg masses were laid with very high synchrony (*R* = 0.79), on average 1 day before the maximum amplitude tide (347°, Omnibus test *p* ≪ .05, Figure 3, Table 2, 3). Hatching occurred approximately 4 days later (90°) with slightly lower synchrony (*R* = 0.60, Figure 3, Table 2, 3). This matches the 4‐day period of development observed in the field (Table 1). Intensity of reproduction was lower during the dry season, synchrony occurred during both seasons, and average timing of reproduction was <1 day later in the dry season than in the wet season (Figure 5). This species showed no difference in the number of egg masses laid or hatched during full or new moons (binomial test (laid): *p* = .55, hatched: *p* = .18, Figure 4). However, a significantly larger number of reproductive events occurred near the time of the smaller spring tides than the larger spring tides (binomial tests: *p* ≪ .001, Figure 6).

#### 3.1.4 *Nerita scabricosta*

*Nerita scabricosta* hatch after an average of 28 days in the field (Collin et al., 2016). The timing of capsule laying and hatching had very low synchrony (*R*_laying_ = 0.15, *R*_hatching_ = 0.19, Figure 3), with capsule laying and hatching occurring throughout the tidal amplitude cycle. Despite this, neither were uniformly distributed across the tidal cycle (Omnibus test *p* < .05). Average timing of the appearance of new, white capsules was during the lowest amplitude tides (179°), and hatching occurred, on average, approximately 2.5 days after the maximum amplitude tide (62°). The large standard deviation and low *R* indicate a very large amount of variation in timing. Reproduction occurred throughout the lunar cycle, with no clear pattern in timing (Figure 4). This species does not reproduce during the dry season, so seasonal comparisons were not possible.

#### 3.1.5 *Siphonaria palmata*

Observations of *S. palmata* masses collected immediately after laying showed development times of 3.0 days (*SD* = 0; *N* = 10). The maximum number of egg masses per tidal amplitude cycle occurred with very high synchrony (*R* = 0.85), 1–2 days after the lowest amplitude tides (average timing = 214°, Omnibus test *p* < .05, v‐test relative to 180° *p* < .05, Figures 2, 3). Based on a 3‐day incubation period, we estimated the peak in hatching to be ~3 days after the minimum amplitude tide (average timing = 255°, v‐test relative to 0° *p* = 1.0, v‐test relative to 180° *p* < .05, Figure 3). Peaks in egg masses occurred approximately 4–5 days before the new and full moons, with estimated peaks in hatching occurring ~1–2 days before the new and full moons, with equal reproductive intensity during new and full moons (Figure 4, binomial test: *p* = 1). There was no evidence that the reproductive peaks were modulated by the differences between the large and small spring tides (Figure 6, binomial test: *p* = .8). No difference in timing was observed between the seasons (Figure 5).

#### 3.1.6 *Natica chemnitzi*

Masses of *N. chemnitzi* collected during times of peak occurrence hatched at most 4–5 days after collection, suggesting that maximum development time is 5–6 days. The maximum number of egg masses per tidal amplitude cycle occurred with very high synchrony (*R* = 0.82) approximately 1.5 days after the minimum amplitude tide (215°, Omnibus test *p* < .05, v‐test relative to 180° *p* < .05, Figure 3). We estimated hatching to occur 3 days before the maximum amplitude tide (average timing = 287°, v‐test relative to 0° *p* = .03, Figure 3), based on the observed 6 days to hatching. Average timing of reproduction occurred ~1 day later in the dry season than in the wet season (Figure 5). This species also reproduced with equal intensity twice per lunar month, with peaks in egg masses occurring approximately 3 days prior to the new and full moon (Figure 4, binomial test: *p* = .85). Hatching was estimated to have occurred near the timing of the new and full moon. There was no evidence that the reproductive peaks were modulated by the differences between the large and small spring tides (Figure 6, binomial test: *p* = .85).

## 4 DISCUSSION

Four of the six species examined here show well‐synchronized cycles in reproduction that are clearly separated by nonreproductive periods. All of these show two reproductive periods per month, supporting the view that these are synchronized with the tidal amplitude cycle rather than the lunar cycle. Larval release occurs during the largest amplitude tides of the tidal amplitude cycle in two of these species, *L. variegata* and *N. chemnitzi*. One other species, *Cerithideopsis californica*, laid eggs during the maximum amplitude tides and hatched near the end of the half of the 2‐week cycle with larger amplitude tides. Only *S. palmata* hatched during the half of the cycle with the smaller amplitude tides, on average, 3 days after the minimum amplitude tide. In *L. variegata*, reproduction was concentrated during the largest spring tides of the month regardless of lunar phase and minimal reproduction was observed during the smaller of the spring tides in the month.

The two species that do not show well‐synchronized cycles in reproduction are those that have the longest development periods. Time to hatching in *Crepidula* cf. *marginalis* ranges from 8.5 days at 28°C to 10.2 days at 23°C in the laboratory (Collin, 2012), and time to hatching in *Nerita scabricosta* averages 24–33 days in the field (Collin et al., 2016). Clear biweekly cycles in crab reproduction have been observed to decrease with increasing latitude and concomitant increases in the duration of reproduction. This suggests that longer developmental periods may make it difficult to precisely match favored hatching times (Morgan, White, McAfee, Gaines, & Schmitt, 2011); however, development times that are linked to this mismatch in crabs are significantly longer (1–3 months) than that of *C*. cf. *marginalis*, but not of *N. scabricosta*.

### 4.1 Are biweekly cycles common in intertidal gastropods?

Monthly or biweekly cycles have been previously reported for only a few marine gastropods, and most of these are littorinids or siphonarids (Tables 4, 5). Among littorinids, and *Littoraria* in particular, there is ample evidence that release of pelagic egg capsules or brooded larvae usually occurs around the spring tides (Table 4). Virtually, all laboratory‐based studies (including this one) show animals releasing in a clear cycle related to the full/new moon or the largest amplitude tides. However, it may be noteworthy that those studies based on observations of capsules and larvae released and collected in the field do not show such clear lunar or tidal amplitude patterns. For example, autocorrelation analysis of abundance of egg capsules of *Nodilittorina lineolata* collected along a Brazilian coast showed no relationship between capsule abundance and tidal height, but did show a close match to a time series of wave heights, with release occurring during rough seas (Bueno, Moser, Tocci, & Flores, 2010). This pattern was interpreted to support a pattern of propagule release that promotes onshore retention of capsules (Bueno et al., 2010). Egg capsules of *Melarapha cincta* and *M. oliveri* collected from tide pools also showed associations with rough weather, as well as high salinity, rather than any obvious lunar cycle (Pilkington, 1971). The only field study to support a relationship between tidal amplitude and propagule release showed that the presence of littorinid capsules in plankton samples coincided with times when actual water levels (as opposed to the predicted levels) exceeded the mean high water spring tide level (Borkowski, 1971). This study was conducted in Florida, which experiences relatively small tides and where weather may significantly impact water height. Unfortunately, limited details were given regarding the sampling scheme, making it difficult to assess the strength of the conclusions. A systematic survey of patterns of littorinid propagule release comparing release time in both the field and the laboratory would help determine whether intrinsic cycles tracking expected tidal height are modified by actual tidal height or wave exposure experienced in the field. Systematic comparisons could also assess the importance of the tidal height of the primary habitant occupied by each species (Berry, 1986a), the magnitude of the tidal amplitudes, and/or the typical wave heights experienced by each species (Bueno et al., 2010).

**Table 4:** Reproductive cycles documented as peak larval or capsule release in littorinid species. Bold highlights results from this study

Species	Location	Monthly cycle	Location	Reference
*Littoraria*				
*L. ardouiniana*	Hong Kong	Spring tides	Laboratory	Ng & Williams (2012)
*L. melanostoma*	Hong Kong	Spring tides	Laboratory	Ng & Williams (2012)
*L. melanostoma*	Malaysia	Spring tide (full moon only)	Laboratory	Berry & Chew (1973)
*L. scabra scabra*	India	Spring tides (full & new moon)	Laboratory	Maruthamuthu & Kasinathan, (1986)
*L. strigata*	Malaysia	Spring tides	Laboratory	Berry (1986a)
*L. angulifera*	Florida	Spring tides with rain	Laboratory	Lenderking (1954)
***L. variegata***	**Panama**	**Spring tides**	**Laboratory**	**This study**
*Littorina*				
*L. littorea*	UK	Spring tides	Field/Laboratory	Fish (1979), Alifierakis & Berry (1980)
*L. planaxis*	California	Spring tides (full and new moon)	Laboratory	Schmitt (1979)
*Melarapha*				
*M. cincta*	New Zealand	None/rough weather	Field	Pilkington (1971)
*M. oliveri*	New Zealand	None/rough weather	Field	Pilkington (1971)
*Nodilittorina*				
*N. millegrana*	Malaysia	Little pattern	Laboratory	Berry (1986a)
*N. pyramidalis*	Malaysia	Spring tides	Laboratory	Berry (1986a)
*N. exigua*	Japan	None/strong waves	Field	Ohgaki (1981)
*N. lineolata*	Brazil	None/wave height	Field	Bueno et al. (2010)
*Peasiella*				
*Peasiella roepstorffiana*	Japan	None/differs between day and night	Field	Ohgaki (1981)

**Table 5:** Reproductive cycles documented as peak number of egg masses observed for *Siphonaria* species. Bold highlights results from this study

Species	Location	Egg deposition	Time to hatch (days)		Reference
Neap tides					
*S. gigas*	Panama	Neap tides		Field	Levings & Garrity (1986)
*S. diemenensis*		Early neap tides	10		Parry (1977)
*S. capensis*	South Africa	Neap tides		Field	Pal et al. (2006)
*S. sirius*	Japan	Neap tides	6–7	Field	Iwasaki (1995)
*S. sihpo*	Japan	Half moon			Abe (1935, 1941 in Iwasaki, 1995)
*S. atra*	Japan	Half moon			Abe (1935, 1941; in Iwasaki, 1995)
*S. japonica*	Japan	Second & fourth quarter moon		Field	Hirano (1980)
*S. baconi*	Australia	Possibly neap tides		Laboratory/Field	Mapstone (1978)
***S. palmata***	**Panama**	**Neap tides**	**3**	**Field**	**This study**
Spring tides					
*S. japonica*	Japan	Full moon/spring tide		Field	Abe (1940 in Hirano 1980)
*S. diemenensis*	Australia	Possibly spring tides		Laboratory/Field	Mapstone (1978)
*S. denticulata*		Spring tides	6	Field	Creese (1980)
No pattern					
*S. tasmanica*	Australia	No pattern (pelagic masses)		Field	Quinn (1983)
*S. pectinata*	Florida	Slight tendency to spring tides	17	Laboratory	Zischke (1974)
*S. alternata*	Florida	No pattern (lecithotrophic larva)	15	Laboratory	Zischke (1974)

Among pulmonate limpets, *Siphonaria gigas*,* S. diemenensis,* and *S. capensis*, all produce egg masses around the neap tides (Levings & Garrity, 1986; Pal, Erlandsson, & Sköld, 2006; Parry, 1977), while *S. denticulata* and *S. pectinata* lay around the spring tides (Creese, 1980; Zischke, 1974; Table 5). There is some evidence that the timing of deposition may vary among populations of the same species. For example, at one site in Japan, *Siphonaria japonica* lays during the second and fourth quarters of the moon (Hirano, 1980), but may deposit eggs only during the full moon at another site (Abe, 1940). Hirano (1980) pointed out that spawning only occurs at night and that periods of low spawning correlate with periods of low nighttime movement induced by the tidal cycle, suggesting that the complex interaction between diurnal cycle, tidal cycle, and patterns of movement may control the pattern of egg deposition.

The timing of hatching can be inferred for three of the species listed in Table 5. *Siphonaria denticulata* in Australia (Creese, 1980) lay their egg masses 2–3 days following the new or full moon and hatch approximately 6 days later, which suggests that hatching occurs around the neap tides. In contrast, Parry (1977) observed that *S. diemenensis* produce egg masses synchronously at the onset of neap tides and that these masses take 10 days to hatch, suggesting that hatching occurs near the spring tides. Likewise, *Siphonaria sirius* lays eggs around the neap tides and they hatch 5–6 days later around the spring tides (Iwasaki, 1995). This variation in projected hatching time further supports the conclusion that reproductive cycles in *Siphonaria* are not all driven by factors selecting for hatching at a particular point in the tidal amplitude cycle. Iwasaki (1995) suggested that egg deposition during neap tides limits emersion of *S. sirius* egg masses during early development, which may increase embryo survival. *Siphonaria gigas* lays egg masses during the neap tides, primarily during the smaller amplitude neaps of a lunar month, and hatching occurs primarily during the smaller amplitude spring tides of a month (Christy, 2013; John Christy, unpublished data). This species is found on exposed rocky shores in the upper midintertidal, where they are submerged by all spring tides. Predation of the unprotected egg masses by fishes during high tide appears to be the primary source of egg mortality. Thus, laying eggs during the lower amplitude neap tides, when full inundation is limited, may minimize risk while allowing larvae to hatch during spring tides (J. Christy, personal communication).

Despite these observations of biweekly cycles, there are numerous studies of reproduction of *Siphonaria* species that make no mention of cycles or synchrony in egg mass appearance. Many of these studies were designed to test for seasonal patterns in reproduction and sampled monthly rather than at the higher frequencies necessary to detect biweekly or monthly cycles. It is therefore unclear whether cycles are more common in *Siphonaria* species than can currently be appreciated from the literature, or whether the species listed in Table 5 are unusual in having such clear synchrony in egg mass deposition. If present, biweekly cycles could have had a significant impact on the results of studies aimed to detect seasonal patterns, if sampling was not at a fixed point in the appropriate cycle.

Clear biweekly reproductive cycles were documented here for a species each of *Cerithideopsis* and *Natica*, genera for which there is no previously published evidence of monthly or biweekly cycles. A single study of a cerithid relative of *Cerithideopsis californica*,* Pirenella cingulata* (as *Cerithidea cingulata*) reported that egg masses were found on the days following the new and full moons (Lantin‐Olaguer & Bagarinao, 2001). Apart from this, synchrony in reproduction remains unreported in these families. Cycles have not been reported in the reproduction of neritids or calyptraeids, and we found no evidence of cycles in the representatives of these families studied here. The single other group for which some comparative data are available, the vetigastropods, shows similar variation among species in how propagule release relates to lunar and tidal amplitude cycles. The trochid *Umbonium vestiarium* spawns around neap tides (Berry, 1986b, 1987), while *Trochus niloticus* spawns at the new moon (Hahn, 1993) and the tropical abalone *Haliotis asinina* spawns just after the full and new moons (Counihan et al., 2001). The presence of larvae of *Melagraphia aethiops, Zediloma atrovirens,* and *Lunella smaragda* in the plankton correlates with storms, and not with tides, lunar cycles, or temperature (Grange, 1976). As most vetigastropods are free‐spawners that likely rely on synchronized reproductive timing to ensure fertilization success, timing in this group may be driven by different factors than in the species studied here, all of which have internal fertilization.

### 4.2 Why do the timing of cycles vary?

Littorinids, snails that release their eggs and larvae directly into the water column, like crabs, most often time their release around the large amplitude tides. This suggests that mother snails are targeting propagule release for the same periods that maximize offshore transport and potentially minimize predation on the larvae or planktonic capsules, as do intertidal crabs. This timing may also be optimal for larval or capsule release as it is the moment when the seawater is closest to the normal habitat of the mother snails, reducing travel costs and predation risk for mothers as well.

Snails that deposit benthic egg masses may face different selection pressures. Timing and location of deposition that ensure survival of embryos to hatching may supersede timing to optimize offshore transport of larvae. Embryos in benthic egg masses may face significant mortality from thermal stress, desiccation, osmotic stress, and predation (Podolsky, 2003; Przeslawski, 2004; Rawlings, 1994). Detailed research on the intertidal bubble snail *Melanochlamys diomedea* shows that timing of egg mass deposition relative to the time of day of the low tides significantly impacts thermal stress experienced by the mass and can therefore be used to predict egg mass survival (Podolsky, 2003). Masses laid during the spring tides are predicted to have significantly higher loss than those that were laid during neap tides. Such strong selection for successful hatching could easily shape synchrony of reproductive timing. It is pertinent to note that both species that do not show clear synchrony in our study are those whose embryos are buffered from external stressors; *C*. cf. *marginalis* brood their embryos, protecting them from predation, desiccation, and osmotic stress, and *N. scabricosta* capsules are well protected from predation with calcium carbonate spherules and are buffered from desiccation by being deposited in pools. *Siphonaria palmata*, which show very clear synchrony, deposit masses in similar pools very slightly lower in the intertidal. They are not so well defended as the capsules of *N. scabricosta*, which suggests that predation during larger amplitude tides may play a role in shaping reproduction around the neap tides. *C. californica*, the species here with the highest exposed egg masses, occurs at a height (~4.25 m) that is always inundated during spring tides, but is at the extreme edge of the high tides during the neap tides; high tides during the smallest high neap tides reach between 3.6 and 4.3 m depending on the month. This suggests that masses that mature much later than average risk being ready to hatch during a period when they are not inundated, or when hatchlings risk being stranded in only a few centimeters of water. Although this does not explain why they reproduce preferentially around the small amplitude spring tides, detailed experimental work and additional comparative studies of related species living at different tidal heights or in different environments are necessary to assess these scenarios before a clear understanding of the factors that influence reproductive synchrony can be reached.
